# Supplementary material for: Strategic management and risk control of emergency hospital construction: SWOT and STPA framework from a systems thinking perspective
Source: PLoS One. 2023 Nov 30;18(11):e0295125. doi: 10.1371/journal.pone.0295125 (PMC10688668; doi:10.1371/journal.pone.0295125)
Supplement: S1 File — (DOCX) [file pone.0295125.s001.docx]

**Technical requirements for the construction of temporary hospitals in Hubei Province**

When Wuhan, Hubei was being hit the hardest by COVID-19, Department of Housing and Urban-Rural Development of Hubei Province collaborated with experts from Central South Architectural Design Institute Co., Ltd. and CITIC General Institute of Architectural Design and Research Co., Ltd, jointly to produce “Technical requirements for the Design and Conversion of Makeshift (FangCang)

Hospitals”(short for MHs). Hopefully, this could be beneficial to the practice of “Leave No One Unattended”. Up to 12,000 patients had been treated in 16 MHs from Feb 3rd 2020 (First MH

put into operation) to March 10th 2020 (All MHs closed). Currently, with the COVID-19 Epidemic spreading quickly around the world, we pulled together professional team again to summarize practice of MH design, construction and operation and amended “Technical requirements for the Design and Conversion of Makeshift (FangCang) Hospitals”. Now, we have it translated into English and issued

to public for peers both at home and around the world to refer to. Hopefully, we join hands to combat this epidemic, overcome trials and tribulations, and solidify our confidence in conquering this major infectious disease endangering all human beings.

## 1 Requirement on building selection for reconstruction

1.1 Building to be reconstructed into MH shall be single storey or multi-storey, while building structure, fire-proof degree, fire prevention section, safe evacuation, fire-fighting facilities and traffic lanes shall be in accordance with currentlocal rule and requireme.

1.2 The site shall be remote from densely populated urban area such as residential area, kindergartens, schools and etc. Also, it shall be remote from storage site of inflammable, explosive, poisonous and harmful gases. Warning board shall be placed outside the MH, while isolated greening strips shall be at least 20 meters between existent building and its surroundings. At least 30 meters shall be remained if there are no greening strips.

1.3 Converted building shall have parking lots and turnarounds in the entrance to allow fast drop-off and pick-up for emergency vehicles. The existent building shall be accessible from the outside while connect well from inside, ensuring basic medical devices, accessible facilities, convenient passenger and materials drop off zones, fully geared security and protection appliances. Temporary houses, tents, mobile testing labs, mobile CT rooms, toilets, bathrooms, and relevant places for sewage treatment shall be available onsite. Buildings with proper equipment and good condition of fire protection such as exhibition center, stadium, big factory, warehouse, dormitory, where internal space can be easily partitioned is highly recommended.

1.4 Masterplan, structure form, height, HAVC, water supply and drainage, power supply and distribution, telecommunication, fire-fighting of the existent building, nearby municipal supporting conditions and indoor facilities should basically meet the requirements demand of MH or at least meet after renovation.

1.5 Building to be converted shall be structurally safe, and can be easily tested for the structure condition. Frame or large span structure is recommended for its convenient indoor dismantle or conversion.

## 2 Requirement on MH Conversion Details

2.1 Conversion details includes: outdoor municipal facilities, sewage treatment facilities, indoor partitions, indoor facilities and appliances, traffic lanes to outside, traffic lanes for people and materials, isolation and improvement of surrounding area, hygiene and pandemic prevention, biosafety, safe prevention and protection and etc. Theoretically speaking, indoor functions and site facilities shall be concerted only.

2.2 Buildings shall be used as MH only from conversion to the completion of being taken over.

2.3 The built MH shall meet the demand of local department of hygiene and health, department of disease control and onsite medical staff.

2.4 If the existent building cannot accommodate clauses in “1. Requirement on building selection for conversion”, it shall be converted appropriately to meet the demand.

## 3 Requirement on architectural plane layout and quarantine sections

3.1 Architectural layout should in compliance of “THREE AREAS AND TWO PASSAGES” (contaminated area, semi- contaminated area, clean area; medical staff passage and patient passage). Medical staff passage shall be separated from patient passage, while clean area shall be separated from contaminated area, negative pressure ventilation system shall be adopted, enough places shall be allocated for plant facilities and patient active area.

3.2 Detailed requirement for “THREE AREAS AND TWO PASSAGES” are as follows, contaminated area includes treatment area for confirmed cases with mild symptoms, area for hospital beds, observation and treatment room, disposal room, medical material disposal room, contaminated material room and rooms for patients to proceed hospital discharge and admission. Clean area includes dressing room, food preparation room, duty room and warehouse. Semi-contaminated area refers to those between clean area and contaminated area, which includes places where could be potentially contaminated by patients’ blood or body fluid, such as medical staff office, treatment room, nurse station, medical device treatment room and internal walking passage and etc. Different colors shall be applied to differentiate contaminated, semi-contaminated and clean area.

3.3 Appropriately design diagnostic and treatment flow. Two rooms shall be placed between clean area and contaminated area, namely entrance and exit passage room. Entrance flow is dressing room I, dressing room II, buffer room, which allows medical staff to put on protective gears before entering contaminated area from clean area. Exit flow is buffer room, protective clothing undressing, buffer room, isolation clothing undressing, bathing, cloth changing, medical staff then enter clean area from contaminated area, but male and female for separate paths.

3.4 Obvious signage or isolation strips shall be available in different areas. Area for hospital beds shall be divided into sections where each section no more than 42 beds. Also, female patients need to be separated from males. Two emergency exits shall be ready for each section, and no more than 30 meters from anywhere in the section to the emergency exit. Firefight evacuation passage shall be available between sections, with width of at least 4 meters in open and broad space. Indicating signage shall be available on the ground of internal passage between section and emergency evacuation passage. Partition materials shall be anti-inflammable with surface can easily cleaned, and height of at least 1.8 meters. Hospital beds equipped with bedside tables shall be parallel for the convenience of medical staff with at least 1.2 meters in between. In case of double-row beds, at least 1.4 meters shall be remained between ends of close beds. In case of single-row beds (very rare circumstance), at least 1.1 meters shall be remained between bed end and wall.

3.5 The numbers of people accommodated by converted building of each floor or open and big space shall be determined by existent evacuation exit width. The net width of evacuation exit shall comply with local firefighting design rules, or base on at least 1 meter of 100 people.

3.6 Toilets for patients and medical staff shall be separated. Patients use temporary toilets while special passage shall be allocated between toilets and area of hospital beds. The foam-sealed mobile toilets are preferred. The number of toilets shall be configured according to the standards of 20 people/squatting position for men's rooms and 10 people/squatting position for women's rooms, and additional more toilets may be allocated according to the actual requirements of patients. The toilets shall be located at the leeward of the buildings and as far away as possible from catering areas and water supply points. The domestic sewage from temporary toilets and other facilities for patients, and bathing wastewater must be disinfected. It is strictly prohibited to directly discharge untreated or substandard ward sewage, medical sewage and ward solid waste. The existing toilets and bathing areas of the buildings are allocated only for healthy medical staff and logistic support workers.

3.7 Accessibility design: The main entrances and exits and internal medical passages shall be equipped with the wheelchair-accessible ramps to all medical departments. The existing internal passages of buildings with a height difference shall be connected with ramps, and the ramps shall be designed according to the accessibility requirements, and in a width necessary for passage of both moving sickbeds and nursing staff.

3.8 Allocation of auxiliary rooms: The entrance for patients shall be equipped with rooms for storage of personal belongings, disinfection and security check, men's lock room and women's locker room, etc. The exit for patients to be transferred to another hospital and for recovered patients shall be equipped with a disinfection and packing area. In addition, the emergency treatment room, treatment room, pantry room, linen room, boiling water room, filth cleaning room and temporary storage room for domestic waste may be allocated near the ward zone (the filth cleaning room and temporary storage room should be close to the exterior wall and the sewage outlet). The medical and nursingclean area may be allocated with infusion preparation room (pharmacy), pharmaceutical warehouse, sterile warehouse, pantry room, rest room for duty staff, office and other rooms.

## 4 Requirements for Structural Safety

The buildings shall be evaluated in the aspect of safety before conversion into MHs, to avoid any potential safety hazards. In the design, under the circumstance that the service load may exceed the original design floor live load, the structural designer shall review the relevant load data and take corresponding measures according to 9 the review results. Focus shall be paid on the following items:

4.1 If heavy medical equipment is allocated, the equipment load information and layout plan shall be reviewed, and corresponding measures shall be taken according to the review results, including no change (the equipment load is less than the design live load), reinforcement or position change (the equipment load is greater than the design live load).

4.2 When partitions are arranged on the floor, the layout plan of partitions and the load information of partition materials shall be reviewed, and corresponding measures shall be taken according to the review results, including no change (the partition load is less than the design live load), reinforcement or replacement with lighter material (the partition load is greater than the design live load).

4.3 When heavy mobile equipment is allocated, the weight of the mobile equipment and movement route shall be reviewed, and corresponding measures shall be taken according to the review result.

4.4 New partitions shall be installed firmly and connected securely.

## 5 Requirements for Fire-fighting Facilities

5.1 The existing fire-fighting facilities shall be in normal operating conditions. It is necessary to ensure emergency evacuation lighting is in normal operating conditions. Ground evacuation signs for zones shall be clearly visible. The existing emergency exits shall meet the requirements and shall be kept clear.

5.2 The corresponding number of fire extinguishers shall be allocated according to the standard for the place with critical hazard level, and the fire extinguishers shall be allocated for the buildings according to the local fire protection rules.

5.3 The rooms for valuable equipment, medical record room and computer room of information center (network), etc. shall be allocated with gas extinguishers.

5.4 If there is no indoor fire hydrant system in the buildings to be converted, fire hose reels or portable fire cocks shall be allocated additionally, and the layout shall meet the requirement that at least one jet of water can be delivered to any part on the same plan.

5.5 Each medical worker in the medical care and medical technology areas shall be allocated with a filtering respiratory protective device for self-rescue from fire, which shall be placed in an eye-catching and easily accessible position in MHs.

5.6 The nurse station should be allocated with a micro fire station, and the mobile high pressure water mist storage should be 100L.

5.7 Where the conditions are available, it shall be ensured that the transformed automatic fire alarm and fire protection linkage control system can operate reliably.

## 6 Requirements for Water Supply and Drainage

6.1 Water supply systems should be equipped with break tanks and disinfecting equipment. Water supply systems should consist of break tanks and pumps. When it is practically difficult to adopt break tanks, analyses of backflow pollution grades of the water supply system should be carried out. Following regulations should be observed.

6.1.1 When the risk of backflow pollution is relatively low and the supply pressure meets the requirements, water supply systems should adopt reduced-pressure type backflow preventers to prevent backflow pollution.

6.1.2 When the risk is relatively high, break tanks should be adopted.

6.2 Water supply pipes and sanitary ware in toilets and bathing areas should not be connected directly, instead there should be air isolation or backflow preventer equipment between them. Certain measures should be adopted in the water supply system to prevent pollution which can be induced by siphon backflow and back-pressure backflow in pipes.

6.3 It is advisable that centralized supply systems with air source heat pumps be adopted for domestic hot water supply in bathing areas. When electric water heaters are adopted, safety devices should be provided.

6.4 Each ward should have its own drinking water supply point which could provide sufficient room temperature direct drinking water and boiled water. Water quality of 13 domestic water should comply with Sanitary Standard for Domestic Drinking Water (GB5749). Bottled water dispensers are acceptable for the supply of boiled water.

6.5 Sewage Treatment. Sewage from temporary portable toilets and wastewater from bathing areas should be disinfected according to General Guidelines of Disinfection in Epidemic Foci (GB19193-2015) and Technical Guidelines for Hospital Wastewater Treatment. Sewage treatment should meet requirements in Standards for Discharge of Wastewater in Medical Institutions. (GB18466) or those in Technical Scheme for Emergency Treatment of Novel Coronavirus-infected Medical Wastewater issued by Ministry of Ecology and Environment before discharge. Faeces, vomit and wastewater from wards should be disinfected before discharge. Solid infected waste and various chemical wastes should not be disposed of and discharged into the sewer. Direct discharge of ward wastewater, medical sewage and ward waste without adequate disinfection which meets certain standards is strictly prohibited.

6.6 Wastewater from temporary outdoor bathing areas should be collected with air-tight piping system and get disinfected before being discharged into the sewage system.

6.7 Condensed water should be collected, discharged indirectly and treated together with wastewater within each zone.

6.8 Washing and disinfecting facilities should be equipped for ambulance parking spots. Washing and disinfecting wastewater should be discharged into the sewage system and the outlets should be treated with water seals.

6.9 All water appliances within the drainage system should either have water seals or be equipped with external water seals. The depth of water seals should not be smaller than 50mm. Safety measures should be adopted to protect water seals from damage. It is strictly prohibited to replace water seals with movable mechanical valves.

6.10 Temporary portable toilets and bathing areas should have respective sewage systems with independent vent pipes.

6.11 It is advisable that rainwater from canopies of temporary outdoor toilets and bathing areas for patients be disinfected before being discharged into the sewage system.

6.12 Plugs should not be used in wash basins.

6.13 Drainpipes should be sealed with unshrinkable, incombustible and non-dusty material. Outlets of vent pipes on drainpipes should be equipped with high efficiency particulate air filters or other reliable disinfection equipment. Draft condition around vent pipe outlets should be good. Outlets of vent pipes on drainpipes should not be connected into exhaust pipes in the HVAC system.

## 7 Requirements for Ventilation and Air Conditioning

7.1 The air inlet and exhaust systems should be set according to the set clean area, semi-polluted area and polluted area. The airflow direction should be from the clean area → semi-polluted area → polluted area according to different pressure gradients. The clean area should be dominated by natural ventilation. When natural ventilation is not available, mechanical ventilation should be provided. Semi-polluted and polluted areas should be dominated by mechanical ventilation.

7.2 When natural ventilation is adopted, the ventilation rate per person should be not less than 226 cubic meters per hour.

7.3 When using separate rooms such as dormitories and hotels to set up isolation wards, the number of air changes in each room should not be less than 6 times / hour. When using isolated spaces in convention and exhibition centers, gyms, and factories to set up isolation wards, the ventilation volume per person should not be less than 150 cubic meters per hour.

7.4 When the original air-conditioning and exhaust system is available, it should be set as a DC-type air supply and exhaust system. The air-conditioning unit closes the return air damper and blocks return air inlet. The outdoor air damper is fully opened, and the outdoor air is sent in. The exhaust air volume should be greater than the supply air volume (if the exhaust fan volume is not enough, you can turn on the smoke extraction fan or increase the exhaust fan), and the junior high efficiency filter is installed at the inlet of the exhaust fan. If the original air-conditioning and exhaust system cannot be used or there is no ventilation system, a mechanical ventilating system should be added. When temporarily installing a mechanical exhaust system, it is advisable to select an air casing with an appropriate air volume and air pressure, 17 set the exhaust outlet to a height not higher than 2 meters, and set up safety protection measures. The ventilation system requires 24 hours uninterrupted operation throughout.

7.5 The setting position of the intake and exhaust air fans (ports) should form a reasonable airflow channel. The airflow process should be short and cover all the ward area to facilitate the rapid discharge of polluted air.

7.6 After the medical staff went from the clean area to the contaminated area, while setting the supply air or system service area no less than 30 times / hour in the "one dressing". D300 short ventilation ducts are set in each adjacent compartment. After the medical staff returned from the contaminated area to the clean area, while setting up the exhaust air which is not less than 30 times / hour or system service area which is not less than 6 times / hour, in the “de-isolated service room”. Each adjacent compartment is provided with a D300 short duct.

7.7 In each isolation ward area, set several air purifiers with sterilization and disinfection functions. Electric heating blankets and several electric oil heaters should be installed in each bed where heating facilities are required.

7.8 Isolation wards shall be provided with toilets and restrooms, and the exhaust air shall meet the ventilation rate of not less than 12 times / hour, and shall be discharged after high-efficiency filtering.

7.9 The installation location of the blower and exhaust fan should be set according to the actual situation. It should be ensured that the fresh air is taken from the outside. The fresh air outlet and the surrounding environment must be clean to ensure that the fresh air is not polluted. The outdoor exhaust should be discharged at a high altitude, and the horizontal distance from any air inlet should not be less than 20 meters, or the vertical distance should not be less than 6 meters.

7.10 The condensate water of the air conditioner in the contaminated area should be collected centrally and discharged into the hospital's sewage drainage system for unified treatment by indirect drainage.

7.11 It is advisable to monitor the alarm signal of the air supply fan and exhaust fan at any time to ensure the normal operation, to monitor the pressure difference alarm of the air filters at various levels of the air supply and exhaust system at any time, and to replace the blocked air filter in time to ensure the air volume of the fan.

7.12 Exhaust high efficiency air filter replacement operators must be self-protected. Dismantled exhaust high efficiency air filters should be disinfected in-situ by professionals, put into safe containers for disinfection, and dispose of them with medical waste.

7.13 In the MH with a large space, where the airflow organization is obviously unfavorable, if it is difficult to add an exhaust outlet, an air purifier with sterilization and disinfection functions should be concentrated set, and an industrial air purifier should be given priority.

7.14 For MHs that can only supply air through the negative pressure of the external door, if the wind speed at the external door is too high, measures such as adding baffles should be adopted to reduce the impact of the wind speed on the patients.

7.15 A certain number of mobile oxygen cylinders should be configured according to the needs to meet the oxygen demand of patients.

## 8 Requirements for Electric and Intelligent Management

8.1 The power supply capacity of the converted building should meet the demand of power load for the new function after conversion. The addition of distribution power lines should meet the requirements of protection sensitivity.

8.2 It is advisable to keep distribution boxes and the controllers of the site away from the contaminated areas and to place them in a special room, if possible.

8.3 It is advisable that standard product packages be used for controllers of ventilation equipment which could be put under the centralized control of nurse stations (duty offices).

8.4 When conditions permit, each bed should be provided with 1-2 220V, 10A single-phase sockets and table lamps; in places with insufficient conditions, multiple groups of single-phase power socket boxes can be set in the surrounding area of the large open room, which is convenient for patients to charge their mobile phones. When required to install electric blankets, they should be equipped with a separate power supply circuit, with a centralized and time-sharing control to reduce fire hazards.

8.5 In order to reduce the glare effect of luminaires on the top of existing building, it is advisable to add some luminaires and pole lights with opaque lamp shade or indirect illumination on the peripheral walls of large-bay areas or on the ground, if circumstances permit.

8.6 Additional luminaires and socket circuits should be equipped with residual current operated protective devices of 30mA.

8.7 Accesses to wireless networks should be provided for the complete coverage of 4G/5G networks. If circumstances permit, a wireless AP should be added to achieve the complete coverage of WIFI.

8.8 Additional ground luminaries, power socket circuits and weak-current circuits should be installed with metal conduits (slots). It is advisable that layout of conduits (slots) should avoid personnel and logistics passageways.Necessary measures should be taken when it’s impossible to avoid them.

8.9 The places which provide medical treatment and necessitate disinfection need to be equipped with sockets for ultraviolet lamps or air sterilizers. Ultraviolet lamps should have identifiable specific switches which could not be parallel to those of ordinary lamps. If ultraviolet lamps are used in places with human presence, it is advisable to use indirect-type lamps or those which have adjustable angles.

8.10 Medical equipment rooms, bathrooms toilets with shower areas should be equipped with auxiliary (local) equipotential bondings.

8.11 Nurse stations (duty offices) should be equipped with one-button alarm system which links with the security and safeguard system. Nurse stations should allow for the power supply of high-power appliances and it is advisable to have two sockets circuits for power supply.

8.12 It is advisable to apply video surveillance systems to patient rest areas and nurse stations.

8.13 When conditions for the wiring of new weak-current and intelligent system do not exist, wireless solutions could be adopted.

8.14 Information management systems should share corresponding information as 23 required by administrative departments.

8.15 Broadcasting and information-distribution systems need to be equipped with public broadcasting systems. It is acceptable to make use of old ones in the original sites and to connect the wiring port into nurse stations. Temporary broadcasting systems which adopt the scheme of distributed networks are also acceptable.

8.16 It is advisable that module hospitals be equipped with multi-parameter vital signs detectors which collect patients’ vital signs and upload records automatically.

8.17 It is advisable that module hospitals be equipped with mobile integrated remote consultation terminals based on conference video.

8.18 It is advisable that accesses for personnel be equipped with non-contact rapid body temperature measurement equipment.

8.19 It is advisable that module hospitals be equipped with disinfection robots and logistics robots which enable various non-contact services.

## 9 Requirements for Site Construction

9.1 Adopt the integrated construction mode of design, procurement, construction and acceptance by integrating the design, procurement and construction when units of design and construction cooperate closely and proceed simultaneously at the construction site.

9.2 The zone division, operation segmentation, and work teams detachment should be carried out during the construction in accordance with the requirements of modularization, standardization and assembly, to avoid cross-work between teams and set reasonable aside time intervals between procedures.

9.3 The partition wall construction shall be organized according to the design requirements of the building plane and quarantine partition layout. The partition wall shall be made of the lightweight fireproof materials. Take the partition unit as the inspection batch, check and inspect the rigidity, strength and stability of the partition wall and the tightness of the joint.

9.4 Special partial strengthening measures shall be taken at such places where pipelines passing through the partition wall and the equipment attached to the partition wall, and the crack prevention measures shall be taken at the junctions of the light partition wall with the ceiling or with other walls.

9.5 Check and test all the relevant indicators of ventilation, air conditioning, and building electricity to ensure that they meet the requirements of design and relevant standards and specifications.

9.6 Strengthen the epidemic prevention and safety management of all on-site operators. Fixed temperature measuring points shall be set at each entrance and exit, and set the mobile temperature measuring personnel to conduct a mobile temperature measurement randomly every four hours. The staff should wear masks correctly to avoid cross-infection. Ventilate the construction site to keep the air flowing. Disinfect the toilets and offices every 6 hours during construction.

9.7 Smoking is strictly prohibited at the construction site. Strengthen the fire safety at construction sites, reduce the open flame operations, and set up fire extinguishers or mini fire stations in accordance with the fire protection requirements.

9.8 The dual-circuit backup power supply should be set up, and the leakage protector should be installed in different zones to ensure the safety of power consumption during construction and operation.

## 10 Other Requirements

10.1 Refuse disposal: Each unit of the ward is equipped with clinical capped garbage bins covered with medical waste garbage bags. Non-medical waste placed in dictated trash bins，and shall be cleaned up daily or instantly. Before dump trash bins，use chlorine-containing disinfectant which contained 500mg/L-1000mg/L available chlorine to spray or pour on the trash until completely wet. After 30 minutes, send the waste to the dedicated clinical waste storage room for centralized storage. Clinical waste and domestic waste produced by patients (regarded as clinical waste) will be unified collected by the hospital administration, then transport to appointed place for harmless treatment by qualified institutions certificated by relevant functional departments.

10.2 The sanitary condition of contemporary toilets in MHs need to be inspected and cleaned up instantly, inspection staff should wear protective suits.

10.3 Layouts of building plans and clean-dirt partition should be prominently displayed, indicated with emergency exit locations and escape routes. Daily management shall ensure that the fire passages are unblocked. Establish linkage mechanism with district fire station, formulate pre-proposal on fire fighting and emergency evacuation. One fire engine is equipped outside the hospital.

10.4 Disinfection: Disinfection focuses on the prevention and control of rats, cockroach and flies. Key areas are storage rooms, garbage dump points, pollutant disposal sites, utility rooms and toilets.

10.5 Strengthen the safety inspection during the process of operation of MHs, any detected risk or hidden dangers must be promptly and properly solved.
